# Moderate-intensity treadmill exercise attenuates osteoporotic bone loss in ovariectomized mice via modulation of gut microbiota and glycerophospholipid metabolism

**DOI:** 10.3389/fphys.2026.1752929

**Published:** 2026-03-24

**Authors:** Xiaochen Meng, Xiuguo Cheng, Longxiao Sun, Chong Wang, Kunyi Huang, Ye Li, Yongjie Yang, Li Gao

**Affiliations:** 1 College of Sport and Health, Shandong Sport University, Jinan, Shandong, China; 2 Department of Health and Physical Education, The Education University of Hong Kong, Tai Po, Hong Kong SAR, China

**Keywords:** gut microbiota, metabolomics, moderate-intensity treadmill exercise, osteoporosis, ovariectomized mice

## Abstract

**Objective:**

This study aimed to determine the key gut microbial taxa and metabolites associated with the effects of moderate-intensity treadmill exercise on bone mass in a murine model of post-ovariectomy osteoporosis, using 16S ribosomal RNA sequencing and metabolomic profiling.

**Methods:**

Female C57BL/6 mice (12 weeks old) were randomly allocated to one of three groups: sham-operated (SHAM), ovariectomized (OVX), and ovariectomized with exercise intervention (OVX-EX) (*n* = 15 per group). The OVX-EX group underwent an 8-week regimen of moderate-intensity treadmill exercise. Bone microarchitecture of the distal femur was assessed using micro-computed tomography. Gut microbiota composition was analyzed through 16S rRNA sequencing, and fecal metabolomic profiles were characterized using liquid chromatography–mass spectrometry. Spearman’s correlation analysis was performed to examine the associations among differential bacterial genera, metabolites, and bone mass indices. GC-MS was used to detect short chain fatty acids in feces, and LC-MS was used to detect plasma choline content.

**Results:**

The OVX-EX mice exhibited significantly improved trabecular bone parameters, including BMD, BV/TV, and Tb.N, along with a marked reduction in the abundance of *Hungatella*. Moderate-intensity treadmill exercise normalized the levels of ten metabolites that were dysregulated following ovariectomy. The abundance of *Hungatella* is significantly correlated with eight metabolites including choline. Pathway enrichment analysis indicated glycerophospholipid metabolism as a key regulatory pathway.

**Conclusion:**

Moderate-intensity treadmill exercise alleviated osteoporotic bone loss in ovariectomized mice, potentially by suppressing the proliferation of *Hungatella* in the gut microbiota and activating glycerophospholipid metabolism to upregulate choline levels, thereby improving bone homeostasis.

## 1 Introduction

Postmenopausal osteoporosis (PMOP) is a metabolic bone disease resulting from estrogen deficiency. Its core pathological feature is that the rate of bone resorption exceeds that of bone formation, leading to deterioration of bone microstructure, decreased bone strength, and increased fracture risk, which severely impairs patients' quality of life and imposes a substantial socioeconomic burden. In recent years, the gut microbiota has emerged as a key regulator of host health and has been closely linked to bone metabolism. Liu et al. (2021) demonstrated that transplanting gut microbiota from rapidly growing children into ovariectomized (OVX) mice significantly improved bone mechanical properties, reduced bone loss, and restored bone microstructure, revealing a novel “gut–bone axis” regulatory mechanism mediated by functional extracellular vesicles derived from gut bacteria. These findings suggest that targeting the gut microbiota may represent a promising strategy for PMOP intervention.

Accumulating evidence from both animal and clinical studies indicates that PMOP is associated with marked alterations in gut microbiota composition, and that such microbial dysbiosis can influence host immunity and bone homeostasis through metabolites such as short-chain fatty acids (SCFAs) (Guo et al., 2023; Greenbaum et al., 2022). For instance, SCFAs have been shown to modulate osteoclast activity indirectly by regulating the balance between Treg and Th17 immune cells (Lorenzo, 2021; Yang et al., 2021). Therefore, identifying interventions that exert anti-osteoporotic effects via gut microbiota modulation holds significant scientific and translational value.

Regular moderate-intensity physical exercise is widely recognized as an effective non-pharmacological strategy for promoting bone health. Exercise not only directly stimulates bone formation through mechanical loading but also indirectly influences bone metabolism by enhancing gut microbial diversity and enriching beneficial bacteria and their metabolites (e.g., SCFAs) (Tartibian et al., 2011; Zhang YW. et al., 2022; Clauss et al., 2021). Moreover, exercise has been shown to regulate bone turnover through multiple signaling pathways, including Meg3/P62/Runx2, Wnt/β-catenin, and RANKL/RANK/OPG (Chen et al., 2021a; Chen et al., 2021b; Tobeiha et al., 2020). However, although the “exercise–gut microbiota–bone axis” has been proposed as a regulatory pathway, its specific targets and underlying molecular mechanisms remain poorly understood. In particular, whether exercise exerts anti-osteoporotic effects by modulating specific bacterial genera and associated metabolic pathways has yet to be systematically elucidated.

Building on previous research, the present study investigates the systemic effects of moderate-intensity treadmill exercise on the gut microbiota composition and fecal metabolic profile in OVX mice, aiming to dissect the potential mechanisms through which exercise improves PMOP from a multidimensional “microbiota–metabolism–bone mass” perspective. We hypothesize that moderate-intensity exercise may ameliorate bone microstructure and delay osteoporosis progression by regulating the abundance of specific bacterial genera and their related metabolic pathways. To test this hypothesis, we integrated 16S rRNA high-throughput sequencing, untargeted metabolomics, and micro-CT imaging to systematically identify core microbial and metabolic signatures associated with exercise intervention and to construct a correlation network linking these signatures to bone parameters. This provides a theoretical foundation for understanding how exercise modulates PMOP via the gut–bone axis.

## 2 Methods

### 2.1 Experimental animals and grouping

Twelve-week-old female C57BL/6 mice (20.61 ± 0.94 g) were obtained from Beijing Vital River Laboratory Animal Technology Co., Ltd. [license number: SCXK (Beijing) 2021-0006]. The animals were housed in the Specific Pathogen-Free (SPF) Animal Experimental Center of Shandong Sport University. Mice were maintained under controlled environmental conditions, including 40%–70% relative humidity, an ambient temperature of 20 °C–26 °C, and a 12-h light/dark cycle (lights on from 09:00 to 21:00). All mice were provided *ad libitum* access to standard laboratory chow and sterilized water. Bedding was replaced every 3 days, and water bottles and cages were sterilized and changed weekly.

### 2.2 Establishment of the ovariectomized mouse model

A total of 45 female C57BL/6 mice (12 weeks old) were randomly allocated into two groups: sham-operated (SHAM, *n* = 15) or ovariectomized (OVX, *n* = 30) group. Bilateral ovariectomy was conducted via dorsolateral incisions in accordance with previously described protocols (Souza et al., 2019). Mice were anesthetized using tribromoethanol (0.2 mL/10 g body weight; Beijing Jitian Biotechnology Co., Ltd.), and the dorsal fur was shaved before surgery. Animals were positioned in the prone position, and the surgical area was disinfected using 75% ethanol. Longitudinal incisions were made at the intersection of 0.5 cm lateral to the vertebral column and 1 cm above the iliac crest on both sides. Following the incision of skin and subcutaneous tissues, the abdominal cavity was accessed, and the ovaries, identified as pink, mulberry-shaped structures, were carefully excised. In the SHAM group, only periovarian adipose tissue was removed. After surgery, incisions were disinfected with povidone-iodine, and all animals received penicillin prophylaxis for three consecutive days.

### 2.3 Treadmill exercise intervention protocol

The ovariectomized mice assigned to the exercise intervention group (OVX-EX, *n* = 15) underwent an 8-week moderate-intensity treadmill exercise regimen (Liu et al., 2024). Before formal training, a 1-week adaptation period was implemented, during which mice ran for 30 min daily at an initial speed of 6 m/min, with a 2 m/min increase every other day at a 0° incline. The formal exercise protocol began at 8 m/min during the first week and increased by 1 m/min weekly thereafter, with a treadmill incline of 25°. Each daily 60-min training session consisted of a 5-min warm-up with gradually increasing speed, 50 min of treadmill running at the target speed, and a 5-min cool-down with progressive speed reduction.

Moderate-intensity exercise was defined as an intensity corresponding to 50%–70% of maximal oxygen consumption (VO_2_max) (Lancha et al., 1995). Studies have shown that exercise protocols similar to that used in the present study can achieve approximately 70% of VO_2_max in female mice, supporting the classification of our regimen as moderate-intensity treadmill exercise (Kemi et al., 2002). Furthermore, according to the WHO Guidelines on Physical Activity and Sedentary Behaviour, adults are recommended to engage in moderate-intensity physical activity for 30–50 min per session, 3–5 times per week, with additional health benefits expected when weekly activity exceeds 300 min (Bull et al., 2020). Thus, the exercise duration employed in this study is comparable to that recommended for moderate-intensity exercise in adult female.

### 2.4 Sample collection

Fecal samples were collected in the morning (06:00–09:00) following completion of the exercise session. Samples were placed in pre-labeled sterile cryotubes, sealed, snap-frozen in liquid nitrogen, and stored at −80 °C until analysis. Following anesthesia, Blood samples were collected from the mice, and plasma was separated and stored at −80 °C until analysis. Femora were harvested, and all surrounding muscles, ligaments, and soft tissues were removed. The right femur was fixed in 4% paraformaldehyde solution (Beijing Aoqing Biotechnology Co., Ltd.) and stored at 4 °C. In addition, the uterus was excised, cleared of surrounding adipose tissue, and similarly preserved in 4% paraformaldehyde.

### 2.5 Assessment of body weight, food intake, and femoral microarchitecture

Body weight was recorded weekly starting 2 weeks after surgery, using an electronic balance at the same time each day for consistency. Daily food consumption was determined by subtracting the remaining chow weight with from the standard allotment of 10 g per mouse each day. Microarchitectural evaluation of the right femur was performed using micro-computed tomography (micro-CT). Imaging parameters included a tube voltage of 90 kV, tube current of 0.09 mA, and a voxel resolution of 0.026 × 0.026 × 0.026 mm^3^. Three-dimensional image reconstructions were performed to evaluate trabecular bone microarchitecture, and quantitative parameters were obtained for structural analysis.

### 2.6 Gut microbiota DNA extraction, sequencing, and bioinformatic analysis

Genomic DNA was extracted from fecal samples using the Mag-Bind Soil DNA Kit (OMEGA Bio-tek, USA). The V3–V4 hypervariable regions of the bacterial 16S rRNA gene were amplified by polymerase chain reaction and verified by electrophoresis on 2% agarose gels. Sequencing libraries were constructed using the Illumina TruSeq Nano DNA LT Library Prep Kit, followed by quality assessment with an Agilent Bioanalyzer 2100 and Promega QuantiFluor system. Sequencing was conducted using the Illumina platform. Representative amplicon sequence variants (ASVs) were identified using QIIME2 software and taxonomically annotated based on the SILVA database (version 138) to characterize gut microbiota composition and structure. To minimize the potential interference of rare ASVs, those with a total abundance of less than 10 across all samples were filtered out. The final average sequencing depth per sample reached 50,000 reads, ensuring robust downstream bioinformatics analyses.

### 2.7 Untargeted fecal metabolomic analysis

Fecal samples were thawed at 4 °C and homogenized in a solvent mixture comprising pre-chilled water, methanol, and acetonitrile (1:2:2, v/v/v). The mixture underwent low-temperature ultrasonication for 30 min, followed by incubation at −20 °C for 10 min. Samples were then centrifuged at 14,000 × g for 20 min at 4 °C. Supernatants were vacuum-dried and reconstituted in 100 μL of acetonitrile:water (1:1, v/v) for liquid chromatography–mass spectrometry (LC–MS) analysis. Chromatographic separation was performed on a Vanquish ultra-high-performance liquid chromatography system equipped with a hydrophilic interaction chromatography column. Mass spectrometric data were acquired using a Q Exactive Orbitrap mass spectrometer in both positive and negative electrospray ionization modes. Differential metabolites were identified based on the following criteria: variable importance in projection (VIP) > 1, fold change >1.5 or <0.667, and *p* < 0.05. Pathway enrichment analysis was conducted using MetaboAnalyst 6.0 (http://www.metaboanalyst.ca/MetaboAnalyst/home.xhtml).

### 2.8 Fecal short-chain fatty acid analysis

Fecal samples were slowly thawed at 4 °C. An appropriate amount of each sample was resuspended in 50 µL of 20% phosphoric acid, followed by the addition of isopropyl ether containing 500 µM internal standard. The mixture was vortexed thoroughly and centrifuged at 14,000 rpm for 20 min at 4 °C. The supernatant was transferred to an autosampler vial for GC–MS analysis (Kukaev et al., 2024). Chromatographic separation was performed using an Agilent DB-FFAP capillary column (30 m × 250 μm × 0.25 µm). Peak areas and retention times were extracted using MSD ChemStation software. Standard curves were constructed to quantify the concentrations of acetic acid, propionic acid, butyric acid, and valeric acid in the samples.

### 2.9 Plasma choline quantification

Plasma samples were slowly thawed at 4 °C. An appropriate volume of each sample was mixed with isotope-labeled internal standard and pre-cooled acetonitrile/water (9:1, v/v). The mixture was vortexed and incubated at 4 °C for 10 min to precipitate proteins. After centrifugation at 14,000 rpm for 15 min at 4 °C, 400 µL of the supernatant was loaded onto an Oasis HLB plate and filtered using a positive pressure manifold. The filtrate was transferred to a 1.5 mL microcentrifuge tube and stored at −80 °C. A mixed standard stock solution was prepared. Chromatographic separation was performed on an Agilent 1290 Infinity UHPLC system, and mass spectrometric analysis was conducted in positive ion mode using a 6500+ QTRAP mass spectrometer (SCIEX) (Zhao et al., 2015). Peak areas and retention times were extracted using Multiquant 3.0.2 software. Metabolites were identified by matching retention times with those of authentic standards.

### 2.10 Statistical analysis

Statistical analyses were performed using SPSS software (version 26.0; IBM Corp., Armonk, NY, United States). All quantitative data are expressed as mean ± standard deviation. Data normality was evaluated using the Shapiro–Wilk test. Nonparametric rank-sum tests were applied to 16S rRNA sequencing and metabolomic datasets, while one-way analysis of variance (ANOVA) with Bonferroni *post hoc* test was performed for normally distributed data. Spearman correlation analysis was used to examine associations among gut microbiota, metabolites, and bone mass parameters. Statistical significance was defined as *p* < 0.05, and high statistical significance as *p* < 0.01. Figures were generated using GraphPad Prism (version 9.0) and R software.

## 3 Results

### 3.1 Moderate-intensity treadmill exercise ameliorated osteoporosis in OVX mice

**FIGURE 1:**
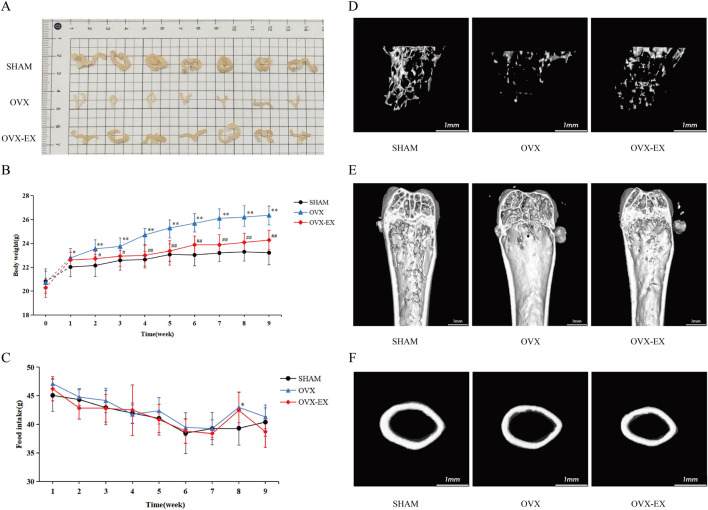
Uterine morphology, body weight, food intake, and femoral micro-CT imaging in SHAM, OVX, and OVX-EX groups. **(A)** Uterine morphology. **(B)** Body weight changes over time. “0” = pre-operation; “1” = adaptation week; “2–9” = weeks 1-8 of formal exercise intervention. **(C)** Average daily food intake. **(D)** Three-dimensional reconstruction of trabecular bone in the femoral medullary cavity. **(E)** Sagittal section of the distal femur. **(F)** Cross-sectional image of femoral cortical bone. *P < 0.05, **P < 0.01 vs. SHAM; #*P* < 0.05, ##*P* < 0.01 vs. OVX. SHAM, sham-operated; OVX, ovariectomized; OVX-EX, ovariectomized with exercise intervention.

Mice in the SHAM group exhibited well-developed uteri with normal tone, whereas the OVX group demonstrated marked uterine atrophy characterized by reduced size and poor tone. The OVX-EX group displayed comparatively preserved uterine morphology and tone compared with the OVX group, indicating that moderate-intensity treadmill exercise partially counteracted ovariectomy-induced uterine atrophy and tone loss. Body weight in the OVX group increased significantly beginning at week 3 post-surgery compared with the SHAM group (*p* < 0.05). Following the 8-week exercise intervention, body weight in the OVX-EX group significantly decreased from week 1 of formal training compared with the OVX group (*p* < 0.05). Food intake remained consistent among all groups (Figures 1A–C).

Micro-computed tomography (micro-CT) analysis of the distal femoral metaphysis (Figures 1D–F; Table 1) demonstrated that ovariectomy resulted in marked deterioration of trabecular bone microarchitecture. Specifically, compared with the SHAM group, the OVX group exhibited increased trabecular separation, decreased trabecular density, and disrupted trabecular network architecture in the distal femoral metaphysis, accompanied by significant reductions in bone microstructural parameters including BMD, BV/TV, and Tb.N (*p* < 0.01). In contrast, the OVX-EX group showed reduced trabecular separation, increased trabecular density, and partial restoration of trabecular structure compared with the OVX group, with significant improvements in BMD, BV/TV, and Tb.N (*p* < 0.05). These findings indicate that ovariectomy induced weight gain and bone loss, whereas moderate-intensity treadmill exercise attenuated bone density reduction and mitigated structural deterioration of the trabecular bone.

**TABLE 1:** Femoral bone microstructural parameters in SHAM, OVX, and OVX-EX groups.

Indicators	SHAM	OVX	OVX-EX
BMD(g/cm^3^)	1.22 ± 0.01	1.19 ± 0.01^**^	1.20 ± 0.01^#^
BV/TV (%)	0.10 ± 0.03	0.04 ± 0.02^**^	0.06 ± 0.02^##^
Tb.Th (mm)	0.08 ± 0.00	0.07 ± 0.01	0.08 ± 0.00
Tb.Sp(mm)	0.28 ± 0.02	0.38 ± 0.03^**^	0.34 ± 0.03^##^
Tb.N (mm^-1^)	2.80 ± 0.17	2.22 ± 0.12^**^	2.45 ± 0.19^##^
Tb.Pf(mm^-1^)	19.42 ± 2.71	26.15 ± 3.00^**^	22.71 ± 1.71^##^
FD	2.20 ± 0.14	1.79 ± 0.13^**^	2.00 ± 0.14^##^
TbCav.CT (HU)	406.09 ± 52.29	275.05 ± 54.26^**^	321.18 ± 30.73^#^

Compared with SHAM.

**means significant *p* < 0.01; Compared with OVX,^#^

means significant *p < 0.05*,^##^

means significant *p* < 0.01.

### 3.2 Moderate-intensity treadmill exercise alleviated gut microbiota dysbiosis in OVX mice

**FIGURE 2:**
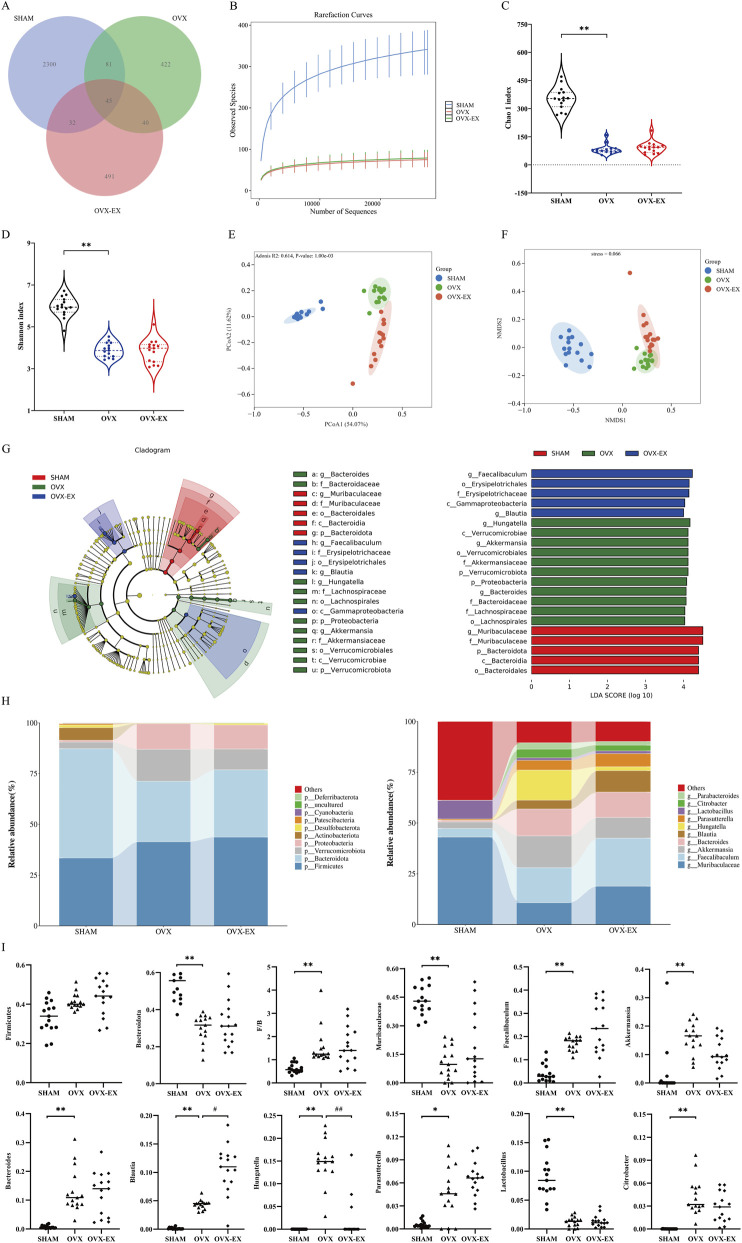
Gut microbiota composition based on 16S rRNA sequencing in SHAM, OVX, and OVX-EX groups. **(A)** Venn diagram showing distribution of ASVs. **(B)** Rarefaction curves. **(C)** Chao1 index. **(D)** Shannon index. **(E)** PCoA based on Bray–Curtis distance. **(F)** NMDS analysis. **(G)** LEfSe results: cladogram and LDA score distribution. Red, green, and blue represent the key microorganisms in the SHAM, OVX, and OVX-EX groups, respectively. **(H)** Relative abundance of top 10 taxa at phylum and genus levels (stacked bar charts). **(I)** Relative abundance at phylum and genus levels (scatter plots). *P < 0.05, **P < 0.01 vs. SHAM; #*P* < 0.05, ##*P* < 0.01 vs. OVX. SHAM, sham-operated; OVX, ovariectomized; OVX-EX, ovariectomized with exercise.

Analysis of 16S rRNA sequencing data (Figures 2A–F) identified 2,458, 588, and 608 ASVs in the SHAM, OVX, and OVX-EX groups, respectively, with 45 ASVs shared among the groups. Unique ASVs totaled 2,300 in SHAM, 422 in OVX, and 491 in OVX-EX groups. Alpha diversity analysis revealed that both the Chao1 richness index and the Shannon diversity index were significantly reduced in the OVX group compared with the SHAM group (*p* < 0.01), indicating decreased microbial richness and diversity following ovariectomy. No significant recovery in these indices was observed in the OVX-EX group after treadmill intervention. Beta diversity analyses, including principal coordinate analysis (PCoA) and non-metric multidimensional scaling (NMDS) based on Bray–Curtis distances, demonstrated distinct clustering among the three groups, indicating differences in gut microbial community composition.

Linear discriminant analyses effect size (LEfSe) (Figures 2G–I) identified 21 taxa with significantly different relative abundances across taxonomic levels, including 5 enriched in the SHAM group, 11 in the OVX group, and 5 in the OVX-EX group. Stacked bar charts of the top 10 taxa at phylum and genus levels demonstrated significant changes in microbial composition among groups. At the phylum level, the Firmicutes/Bacteroidota ratio was significantly increased (*p* < 0.01), and Bacteroidota abundance was significantly decreased (*p* < 0.01) in the OVX group compared with the SHAM group, with no significant phylum-level differences between the OVX-EX and OVX groups. At the genus level, the OVX group displayed reduced relative abundances of *Muribaculaceae* and *Lactobacillus* (*p* < 0.01), and increased abundances of *Faecalibaculum* and *Bacteroides* (*p* < 0.01) relative to the SHAM group. Following treadmill intervention, the OVX-EX group exhibited significantly increased *Blautia* abundance (*p* < 0.05) and markedly decreased *Hungatella* abundance (*p* < 0.01) compared with the OVX group. These findings indicate that ovariectomy substantially disrupted gut microbial composition and diversity, while moderate-intensity treadmill exercise selectively modulated specific bacterial genera, particularly *Blautia* and *Hungatella*, in ovariectomized mice.

### 3.3 Moderate-intensity treadmill exercise regulated metabolic homeostasis in OVX mice

**FIGURE 3:**
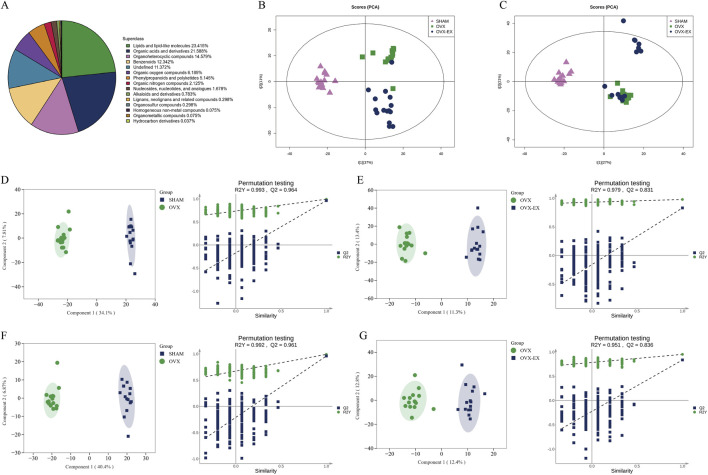
Fecal metabolomic profiles in SHAM, OVX, and OVX-EX groups. **(A)** Classification of detected metabolites by chemical type. **(B,C)** PCA score plots in positive and negative ionization modes. **(D,E)** OPLS-DA score and model validation plots comparing SHAM vs. OVX and OVX vs. OVX-EX in positive ion mode. **(F,G)** OPLS-DA score and validation plots for SHAM vs. OVX and OVX vs. OVX-EX in negative ion mode. SHAM, sham-operated; OVX, ovariectomized; OVX-EX, ovariectomized with exercise.

Untargeted metabolomic analysis identified a total of 2,682 metabolites, comprising 1,679 in positive ion mode and 1,003 in negative ion mode. Chemical classification revealed that lipids and lipid-like molecules accounted for 23.42% of all metabolites, followed by organic acids and derivatives (21.59%), organic heterocyclic compounds (14.58%), and benzenoids (2.34%). Principal component analysis (PCA) and orthogonal partial least-squares discriminant analysis (OPLS-DA) revealed distinct separation between groups. To avoid overfitting during model construction, permutation testing was performed to validate the model. As the permutation retention decreased, the R^2^Y and Q^2^ values of the permuted models progressively declined, indicating good model robustness. These results confirm that the models were stable and that the metabolic profiles were significantly distinct (Figure 3).

**FIGURE 4:**
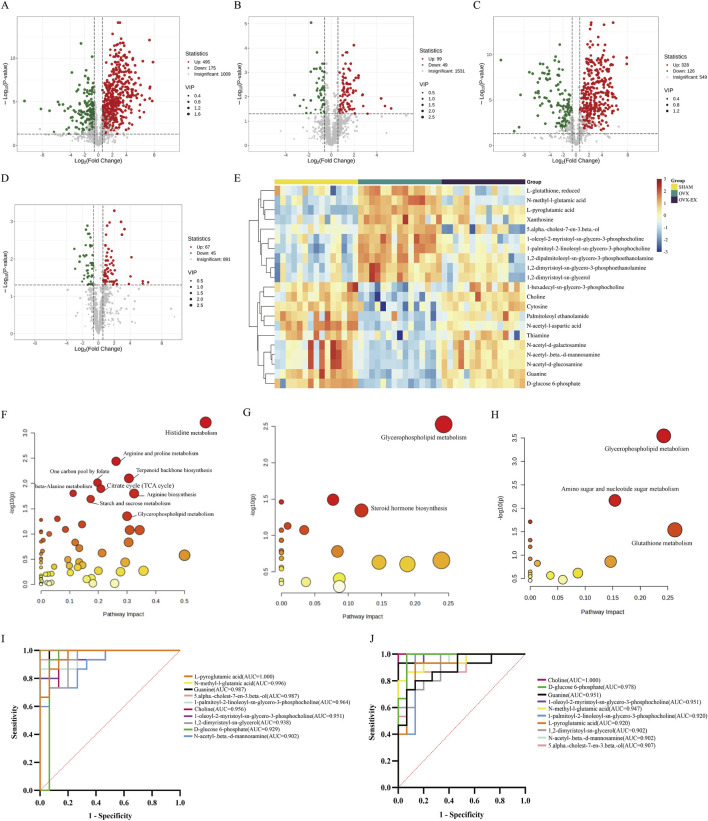
Differential metabolite screening and pathway enrichment analysis. **(A–D)** Volcano plots of differential metabolites: **(A)** SHAM vs. OVX in positive ion mode; **(B)** OVX vs. OVX-EX in positive ion mode; **(C)** SHAM vs. OVX in negative ion mode; **(D)** OVX vs. OVX-EX in negative ion mode. **(E)** Heatmap of differential metabolites across all groups. Groups names are shown on the horizontal axis, and differential metabolites on the vertical axis. Color intensity corresponds to metabolite abundance, with darker colors denoting greater abundance. **(F,G)** KEGG pathway enrichment in positive and negative ion modes. **(H)** KEGG pathway analysis of exercise-responsive metabolites. **(I,J)** ROC curve analysis for SHAM vs. OVX and OVX vs. OVX-EX comparisons.

Comparative metabolite analysis (Figure 4) revealed significant metabolic alterations among groups. In the OVX compared with the SHAM group, 495 metabolites were upregulated and 175 were downregulated in positive ion mode, while 328 were upregulated and 126 were downregulated in negative ion mode. Following treadmill intervention, the OVX-EX group displayed 99 upregulated and 49 downregulated metabolites in positive ion mode, and 67 upregulated and 45 downregulated metabolites in negative ion mode relative to the OVX group. Screening for metabolites exhibiting opposite trends between OVX versus SHAM and OVX-EX versus OVX groups identified 16 differential metabolites in positive ion mode and 5 in negative ion mode. Pathway enrichment analysis using MetaboAnalyst 6.0 based on the Kyoto Encyclopedia of Genes and Genomes (KEGG) database revealed 9 major pathways differentially regulated between SHAM and OVX groups, including 2 identified in negative ion mode. Among the 21 metabolites exhibiting reversed trends after exercise intervention, glycerophospholipid metabolism emerged as a consistently enriched pathway across all three comparison groups, indicating its central regulatory role in exercise-mediated modulation of the osteoporotic phenotype. Receiver operating characteristic (ROC) curve analysis demonstrated that 10 of the 21 differential metabolites achieved areas under the curve (AUC) values exceeding 0.9 (Table 2), indicating strong discriminatory potential as biomarkers for exercise-induced metabolic modulation in osteoporosis.

**TABLE 2:** Ten differential fecal metabolites identified through ROC analysis.

No	Compounds	Ion mode	OVX vs. SHAM			OVX-EX vs. OVX		
			VIP	FC	Trend	VIP	FC	Trend
1	1-Oleoyl-2-myristoyl-sn-glycero-3-phosphocholine	Pos	1.345	2.558	↑^**^	2.319	0.405	↓^##^
2	1-Palmitoyl-2-linoleoyl-sn-glycero-3-phosphocholine	Pos	1.428	2.758	↑^**^	2.237	0.508	↓^##^
3	1,2-Dimyristoyl-sn-glycerol	Pos	1.307	2.179	↑^**^	2.035	0.519	↓^##^
4	5.alpha.-cholest-7-en-3.beta.-ol	Pos	1.474	2.279	↑^**^	1.918	0.470	↓^##^
5	Choline	Pos	1.283	0.516	↓^**^	2.447	1.992	↑^##^
6	Guanine	Pos	1.459	0.284	↓^**^	2.497	2.630	↑^##^
7	N-acetyl-.beta.-d-mannosamine	Pos	1.093	0.242	↓^*^	2.417	2.789	↑^##^
8	N-methyl-l-glutamic acid	Pos	1.486	4.489	↑^**^	2.472	0.287	↓^##^
9	D-glucose 6-phosphate	Neg	1.266	0.136	↓^**^	2.473	4.023	↑^##^
10	L-pyroglutamic acid	Neg	1.490	10.939	↑^**^	1.715	0.507	↓^##^

Arrows indicate relative changes: ↑ indicates an increase; ↓ indicates a decrease. Statistical significance was adjusted using false discovery rate (FDR) correction: *p < 0.05 **p < 0.01 vs. SHAM; *##p* < 0.01 vs. OVX.

### 3.4 Correlation analysis among gut microbiota, metabolites, and bone parameters

**FIGURE 5:**
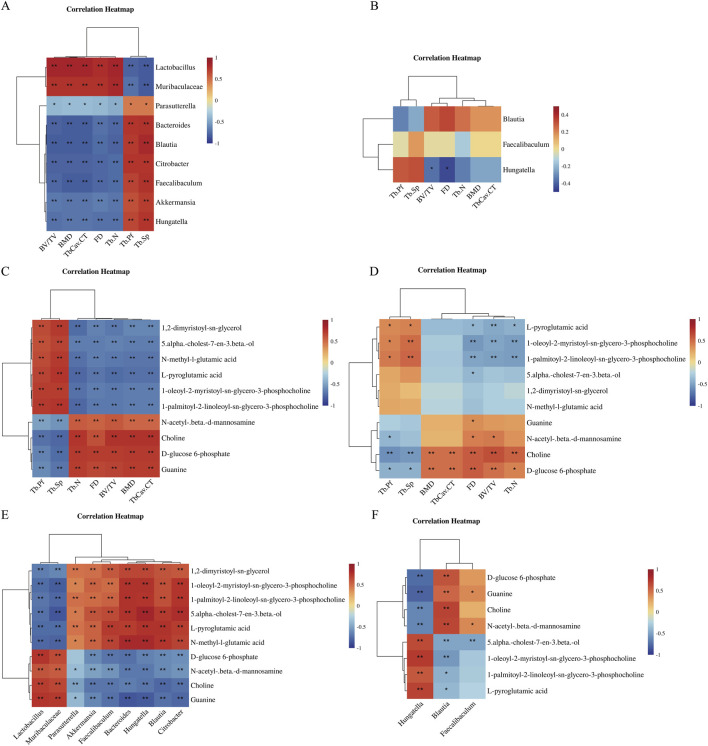
Correlation analyses among gut microbiota, fecal metabolites, and bone parameters. **(A,B)** Spearman correlations between differential bacterial genera and bone structural parameters for SHAM vs. OVX and OVX vs. OVX-EX. **(C,D)** Correlations between differential metabolites and bone parameters for SHAM vs. OVX and OVX vs. OVX-EX. **(E,F)** Correlations between differential metabolites and bacterial genera for SHAM vs. OVX and OVX vs. OVX-EX. *P < 0.05, **P < 0.01. SHAM, sham-operated; OVX, ovariectomized; OVX-EX, ovariectomized with exercise.

Spearman correlation analysis identified specific associations between gut microbial genera and bone structural indices (Figure 5). Following ovariectomy, *Lactobacillus* and *Muribaculaceae* demonstrated strong positive correlations with bone mineral density (BMD), trabecular number (Tb.N), and bone volume fraction (BV/TV) (*p* < 0.01), whereas *Blautia* and *Faecalibaculum* exhibited negative correlations with these parameters (*p* < 0.01). After treadmill exercise intervention, *Hungatella* remained negatively correlated with trabecular bone density and BV/TV (*p* < 0.05).

Metabolite–bone parameter correlation analysis indicated that choline and D-glucose 6-phosphate were positively correlated with BMD, Tb.N, and BV/TV in the OVX group (*p* < 0.01). Conversely, 1-oleoyl-2-myristoyl-sn-glycero-3-phosphocholine and 1-palmitoyl-2-linoleoyl-sn-glycero-3-phosphocholine demonstrated negative correlations with these bone parameters (*p* < 0.01 or *p* < 0.05). These patterns persisted after exercise intervention, with choline and D-glucose 6-phosphate maintaining positive associations (*p* < 0.01 or *p* < 0.05), while phosphocholine derivatives retained negative correlations with Tb.N and BV/TV (*p* < 0.01). Microbiota–metabolite correlation analysis indicated that *Hungatella*, *Blautia*, and *Bacteroides* were positively correlated with 1-palmitoyl-2-linoleoyl-sn-glycero-3-phosphocholine and 1-oleoyl-2-myristoyl-sn-glycero-3-phosphocholine (*p* < 0.01), and negatively correlated with choline and D-glucose 6-phosphate (*p* < 0.01). Conversely, *Lactobacillus* and *Muribaculaceae* demonstrated opposite correlation patterns. After exercise intervention, *Hungatella* continued to demonstrate positive correlations with 1-oleoyl-2-myristoyl-sn-glycero-3-phosphocholine and 1,2-dimyristoyl-sn-glycerol (*p* < 0.01), and negative correlations with choline and D-glucose 6-phosphate (*p* < 0.01). Notably, *Hungatella* displayed significant correlations with eight metabolites related to bone mass, particularly choline and its derivatives. Collectively, these results indicate that moderate-intensity treadmill exercise mitigated bone loss in ovariectomized mice by partially restoring gut microbial balance and regulating metabolite levels, with *Hungatella* and choline-related metabolites identified as potential key mediators of exercise-induced bone protection in PMOP.

### 3.5 Moderate-intensity treadmill exercise increased plasma choline levels

As shown in Table 3, compared with the SHAM group, the OVX group exhibited significantly decreased levels of acetic acid, propionic acid, butyric acid, and valeric acid (*p* < 0.01 or *p* < 0.05). However, no significant differences in these SCFAs were observed between the OVX-EX and OVX groups (*p* > 0.05). In addition, plasma choline levels were significantly lower in the OVX group than in the SHAM group (*p* < 0.05), whereas they were significantly restored in the OVX-EX group compared with the OVX group (*p* < 0.05).

**TABLE 3:** The contents of fecal SCFAs and plasma Choline in SHAM, OVX, and OVX-EX groups.

Metabolites	SHAM	OVX	OVX-EX
Acetic acid	3312.39 ± 1185.36	2082.94 ± 525.97^**^	2038.29 ± 638.34
Propionic acid	315.34 ± 113.93	211.32 ± 49.93^*^	277.43 ± 141.81
Butyric acid	285.54 ± 286.15	7.53 ± 8.83^**^	20.71 ± 51.94
Valeric acid	30.36 ± 23.15	3.20 ± 1.68^**^	5.66 ± 4.43
Choline	2547.25 ± 279.39	2222.26 ± 236.94^*^	2557.97 ± 220.54^#^

Compared with SHAM.

^**^means significant *p* < 0.01; Compared with OVX,^#^

means significant *p < 0.05*,^##^

means significant *p* < 0.01.

## 4 Discussion

### 4.1 Moderate-intensity treadmill exercise modulated gut microbiota composition in ovariectomized mice

The marked decline in estrogen levels following menopause results in a range of physiological alterations, including increased adiposity, loss of muscle mass, lipid metabolism dysfunction, and decreased BMD. In the present study, OVX mice demonstrated significant weight gain and bone deterioration, whereas treadmill exercise effectively mitigated these changes, consistent with our prior findings (Yang et al., 2022; Gao et al., 2021). Importantly, food intake remained comparable among all groups, aligning with results reported by Kawao et al. (Kawao et al., 2021; Chou et al., 2022). These findings indicate that alterations in postprandial metabolic processing, potentially mediated by gut microbial modulation, contributed to the observed differences in metabolic and skeletal outcomes.

Exercise-induced mechanical loading contributes to bone remodeling and improves bone geometry and strength. By activating mechanosensitive signaling pathways in bone tissue, exercise effectively inhibits osteoclast activity and promotes osteoblast differentiation (Zhang L. et al., 2022). In addition, enhanced muscle contraction during exercise stimulates the release of bioactive factors such as insulin-like growth factor 1 (IGF-1) and osteocalcin, thereby reinforcing the endocrine crosstalk between bone and muscle and optimizing systemic metabolic homeostasis (Zhang S. et al., 2022). Vasto et al. demonstrated that a 20-week SuperJump exercise program improved insulin metabolism via the gut hormones GLP-1 and GIP, identifying these peptides as key mediators of exercise-induced benefits on bone health and glycemic homeostasis (Vasto et al., 2022).

Both *Muribaculaceae* and *Lactobacillus* are recognized as beneficial bacterial taxa critical for maintaining intestinal and systemic metabolic equilibrium because they are associated with the regulation of glucose and lipid metabolism (Zhu et al., 2024). Increasing evidence supports the regulatory influence of the gut microbiota in bone homeostasis. The current study identified reduced Chao1 and Shannon indices in OVX mice compared with SHAM controls, indicating diminished microbial richness and diversity following estrogen depletion. Wang and Zhao et al. (Wang et al., 2022a; Zhao et al., 2019) consistently observed reduced α-diversity in both ovariectomized rats and postmenopausal women with osteoporosis, confirming that steroid deficiency-induced gut dysbiosis is an important component of the pathophysiology of osteoporosis. The present study observed alterations in the structure and composition of the gut microbiota, as well as decreased short-chain fatty acid levels, in ovariectomized mice with osteoporosis, suggesting a close association between the pathogenesis of PMOP and the gut microbiota and its metabolites.

As an environmental modulator, exercise can alter the composition of the gut microbiota, increase the abundance of beneficial bacteria, and restore microbial homeostasis, thereby promoting overall health. Multiple studies have confirmed an association between physical activity and gut microbial structure (Evans et al., 2014; Clarke et al., 2014). For instance, Morita et al. (Morita et al., 2019) demonstrated that 12 weeks of aerobic exercise increased the abundance of *Bacteroides* and improved cardiorespiratory fitness in healthy older women, supporting the role of exercise in optimizing the gut microbial profile. In the current study, treadmill exercise significantly reduced the relative abundance of *Hungatella* in the OVX-EX group compared with the OVX group. *Hungatella* exhibited negative correlations with trabecular bone density and BV/TV. Sarcopenia and osteoporosis, both age-related disorders with overlapping metabolic and inflammatory etiologies, have been linked to specific microbial signatures. Prior studies have identified positive associations between the abundance of *Hungatella* and sarcopenia severity, with elevated levels of *Hungatella* observed in individuals over the age of 60 years (Wang Y. et al., 2022; Victoria et al., 2022). *Hungatella* is known to produce trimethylamine (TMA), a precursor of trimethylamine N-oxide (TMAO), which activates NF-κB signaling and enhances osteoclast-related gene expression, contributing to accelerated bone resorption and osteoporosis progression (Rehman et al., 2023; Wang et al., 2022c). These findings indicate that over proliferation of *Hungatella* may impair bone homeostasis through inflammatory and metabolic mechanisms. In this context, moderate-intensity treadmill exercise might exert osteoprotective effects by suppressing *Hungatella* abundance, thereby restoring microbial balance and alleviating bone deterioration in OVX mice.

### 4.2 Moderate-intensity treadmill exercise regulated fecal metabolic profiles in ovariectomized mice

The gut microbiota mediates host–environment interactions may through the production of bioactive metabolites, many of which serve as critical regulators of skeletal homeostasis and reflect functional microbial activity. In the present study, ten key metabolites were identified as potential biomarkers of exercise-induced metabolic regulation in OVX mice, including three choline-associated compounds. Choline is a key component of neurotransmitter acetylcholine, which contributes to bone formation and is a critical participant in lipid metabolism (Wiedeman et al., 2018; Kauschke et al., 2015). In the present study, plasma choline levels were found to be decreased in OVX mice, whereas they were significantly increased in the OVX-EX group. Prior studies have shown that reduced choline levels are associated with increased susceptibility to osteoporosis in older adults (Li Q. et al., 2022). A cross-sectional investigation demonstrated that individuals receiving regular choline supplementation exhibited higher BMD, with choline levels positively correlating with bone density (Runting et al., 2024). Phosphocholine is a key activated intermediate in the choline metabolic pathway and is generated via phosphorylation of choline catalyzed by choline kinase. Song et al. identified phosphocholine as a critical serum biomarker in OVX mice and observed elevated levels of 1-palmitoyl-sn-glycero-3-phosphocholine (lysophosphatidylcholine, LPC) following ovariectomy (Song et al., 2023). Consistent with these findings, the present study found increased LPC levels in OVX mice, which were attenuated after moderate-intensity treadmill exercise. Palmieri et al. reported that phosphocholine oxidation adversely affected murine bone mass and could represent a metabolic risk marker for osteoporosis (Palmieri et al., 2025).

This study found that moderate-intensity treadmill exercise increased plasma choline levels, while no significant changes were observed in the levels of short-chain fatty acids (acetic acid, propionic acid, butyric acid, and valeric acid). These findings suggest that detecting plasma metabolite markers such as choline may help predict bone loss and osteoporosis severity in postmenopausal women, as well as the intervention effects of exercise. Lipidomic profiling by Aleidi et al. identified phosphatidylcholines as the most dysregulated lipid class in individuals with low BMD (Aleidi et al., 2022). Silva et al. demonstrated that individuals participating in high levels of aerobic exercise, such as swimmers, exhibited the lowest plasma concentrations of phosphatidylethanolamines, phosphatidylcholines, and phosphatidic acids (Silva et al., 2024). Dietary components such as choline and phosphatidylcholine can be metabolized by bacteria from the Firmicutes and Proteobacteria to produce trimethylamine (TMA), which is subsequently converted into trimethylamine N-oxide (TMAO) by hepatic enzymes. Elevated TMAO levels accelerate bone loss during the progression of osteoporosis, whereas antibiotic-mediated microbial suppression significantly reduces circulating TMAO levels (Tang et al., 2013). Therefore, regulating gut microbial composition to suppress TMAO formation may represent a promising strategy for PMOP mitigation. The observed correlations between *Hungatella* and choline-related metabolites in this study indicate that exercise may suppresses *Hungatella* proliferation, thereby limiting TMAO biosynthesis from choline derivatives.

Estrogen deficiency is known to disrupt the balance between osteogenesis and adipogenesis, which can contribute to increased adiposity and decreased bone formation. The present metabolomic analysis revealed that most differential metabolites belonged to lipid and organic acid classes, with enrichment in glycerophospholipid metabolism pathways. These findings indicate that lipid metabolic imbalance is closely associated with bone loss and that glycerophospholipid metabolism may constitute a central regulatory mechanism in skeletal maintenance. Glycerophospholipid pathway activity has been shown to influence bone remodeling by modulating macrophage function, enhancing bone regeneration, and preventing resorption, thereby preserving bone integrity (Toita et al., 2024; Li M. et al., 2022). Wei et al. reported upregulated glycerophospholipid metabolism in OVX mice, identifying it as a critical potential contributor to osteoporosis pathogenesis (Wei et al., 2021). Hintikka et al. identified glycerophospholipid metabolism as the most significantly enriched fecal metabolic pathway following 6 weeks of endurance exercise in overweight women (Hintikka et al., 2023). Collectively, these findings indicate that modulation of glycerophospholipid metabolism represents a principal mechanism through which exercise enhances skeletal health and mitigates osteoporotic progression. Lipid metabolism dysregulation is a key pathological mechanism in PMOP (Zhang et al., 2023), with marked changes in glycerophospholipids (Liu et al., 2025). Choline-involved glycerophospholipid metabolism has been consistently identified as a major differential pathway in osteoporosis metabolomics studies (Wei et al., 2021; Zhang et al., 2023). Moreover, choline-related metabolites may regulate the Wnt/β-catenin pathway in BMSCs to promote osteogenic factor expression (Wang et al., 2020). Together, these findings suggest that moderate-intensity treadmill exercise may alleviate bone loss by modulating gut microbiota colonization, glycerophospholipid metabolism, and choline metabolism.

In this study, using ovariectomized mice as a model, we integrated 16S rRNA sequencing, fecal untargeted metabolomics, and micro-CT analysis to elucidate the effects of exercise intervention from the perspective of the “microbiota-metabolite-bone mass” axis. A core regulatory pathway involving the differential *Hungatella*, glycerophospholipid metabolism, and choline was identified, providing specific candidate microbial taxa and metabolic pathways through which exercise may ameliorate osteoporosis. These findings offer valuable insights for future research. Nevertheless, several limitations should be acknowledged. The use of ovariectomized mice, while a well-established model, may not fully recapitulate human physiology, and thus the translational relevance of our findings to clinical applications requires further validation. Additionally, although spearman correlation analysis revealed associations between gut microbiota, metabolites, and bone parameters, confirmatory experiments such as microbiota transplantation (e.g., *Hungatella* colonization) or metabolite intervention (e.g., glycerophospholipid metabolite supplementation) were not performed. Therefore, the results need further verification.

## 5 Conclusion

The present study demonstrates that gut microbiota and their associated metabolites are closely linked to bone structural parameters. Moderate-intensity treadmill exercise may attenuate osteoporosis in ovariectomized (OVX) mice by suppressing *Hungatella* levels and modulating glycerophospholipid metabolism to upregulate choline. These findings highlight the potential of exercise as a non-pharmacological intervention for PMOP, possibly through *Hungatella*–glycerophospholipid–choline–bone axis. Further research is warranted to elucidate the underlying molecular mechanisms of this axis and to develop effective strategies for the prevention and management of PMOP.

## Data Availability

The 16S ribosomal RNA sequencing data presented in the study are deposited in the National Center for Biotechnology Information(NCBI) repository, accession number PRJNA1435915. Untargeted fecal metabolomic data presented in the study are deposited in the metaboLights repository, accession number MTBLS14039.
